# Ethical Imperatives for Working With Diverse Populations in Digital Research

**DOI:** 10.2196/47884

**Published:** 2023-09-18

**Authors:** Jonathan Herington, Kay Connelly, Judy Illes

**Affiliations:** 1 Department of Health Humanities and Bioethics University of Rochester Rochester, NY United States; 2 Department of Informatics Indiana University Bloomington, IN United States; 3 College of Engineering Michigan State University Lansing, MI United States; 4 Neuroethics Canada, Division of Neurology Department of Medicine University of British Columbia Vancouver, BC Canada

**Keywords:** digital health research, justice, research ethics, diversity, engagement, research participants, participatory

## Abstract

Digital research methodologies are driving a revolution in health technology but do not yet fully engage diverse and historically underrepresented populations. In this paper, we explore the ethical imperative for such engagement alongside accompanying challenges related to recruitment, appreciation of risk, and confidentiality, among others. We critically analyze existing research ethics frameworks and find that their reliance on individualistic and autonomy-focused models of research ethics does not offer adequate protection in the context of the diversity imperative. To meet the requirements of justice and inclusivity in digital research, methods will benefit from a reorientation toward more participatory practices.

## Introduction

In the past 2 decades, there has been a rapid increase in human subject research that uses digital research methodologies. We define digital research as human subjects research through which data are collected by electronic devices such as laptops, mobile devices, wearables, pervasive sensors, digital cameras, and other emerging technologies. This includes research that involves the direct collection of digital data for research only, the parallel collection of data for service provision with research as an ancillary purpose, as well as so-called secondary research that repurposes data (eg, from a biometric watch) originally collected for nonresearch purposes. These digital research methods often yield data used to train, test, and validate machine learning models, and thus are at the heart of questions about the accuracy, transparency, and fairness of machine learning systems in health care and elsewhere [1-4]. Calls to diversify the data sets upon which machine learning systems are trained imply a call to diversify the populations that participate in digital research.

In this paper, we explore the ethical dimensions of conducting digital research with and for diverse research populations. To begin, we make an important distinction between marginalized and diverse populations. We define marginalized populations as research populations or subpopulations that are wholly or mostly comprised of people from communities that have historically been exposed to special risks such as socioeconomic or health threats or to which researchers have special obligations, owing to differential power dynamics or historical instances of research exploitation. We define diverse research populations as those that include participants of different ages, genders, races, ethnicities, religions, incomes, literacies, educational backgrounds, languages, cultural norms, and disabilities. Different kinds of diversity will be relevant in different contexts. Broadly representative research populations include many different kinds of diverse subpopulations with sufficient participants from each population so as to proportionately reflect the demographics of the overall population, even if it is impossible to capture all attributes without a very large study population.

Prior work in research ethics has often focused on the special ethical risks and responsibilities that arise in the conduct of research with people who are members of marginalized populations or otherwise vulnerable. For example, genomics research with indigenous communities has long been held to pose special risks to individuals and communities alike and requires enhanced protocols for collaboration, cultural competency, transparency, and community capacity building [5]. Neuroscience research has followed suit as perspectives on the brain and mind extend well beyond western concepts [6,7], and equitable access to translational interventions remains unrealized [8-10]. Collaborative approaches to research, including participatory action methods toward the cocreation of studies and knowledge sharing have been developed in response. The structured guidance offered by The First Nations principles of “ownership, control, access, and possession” (commonly known as OCAP) and the Collective benefit, Authority to control, Responsibility, and Ethics (CARE) Principles of Indigenous Data Governance of the Global Indigenous Data Alliance [11,12] are 2 examples. Likewise, research on health monitoring tools for patients with rare diseases requires balancing their privacy and unique needs [13].

In this paper, we explore the suitability of conventional human subject protections [14] for dealing with the special challenges of research populations with a wide range of interests, cultures, and capacities. Respect for persons, beneficence, and justice have always been basic principles of research with human subjects [14], but the latter, long neglected among the 3, must now become an equal imperative [15-17]. We begin by revisiting the ethical justification for creating diverse and broadly representative research populations in digital research. Next, we identify the different kinds of ethical risks that researchers must address in the context of digital research, including diverse capacities, cultures, risks, and understandings of technology. Finally, we critically analyze the capacity of existing research ethics frameworks to deal with these issues and provide recommendations for improving the capacity of digital researchers to build just and sustainable relationships with diverse research populations. We conclude that the inclusion of marginalized communities is a facet of justice that is especially heightened in the context of digital research, where the range of experiences with, access to, and meaningfulness of digital technologies varies widely [18].

## Imperative for Diverse Research Populations

The ethical responsibility to include diverse research populations in digital research is grounded in a commitment to ensure both the social value of research is widely realized [19,20] and that there is a fair distribution of the burdens and benefits of the research [16]. This obligation attaches to all research, but in the context of digital research, there are at least 3 reasons why realizing these values require a special emphasis on diverse populations.

First, digital research fuels the production of algorithmic tools that have long been accused of biases that differentially distribute opportunities and harms in diverse populations [21-23]. Many of these harms are due to sampling bias in training sets [24]. Others are due to differential measurement bias for some subpopulations, where methods of collection or proxy variables produce more measurement error for some groups than others [2,25]. A prominent example of this is bias in the accuracy of wearables for users with dark skin tones [26,27]. Carefully diversifying data sets in a way that fairly represents the underlying constructs within every subpopulation is thus crucial to remediating these biases in algorithmic tools.

Second, digital research often relies upon convenience samples or secondary data sets that capture existing users of the collection technology. This has the effect of skewing data toward individuals who are White, wealthy, young, and healthy. For instance, studies of patterns of bicycle trail usage may use deidentified data from the Strava app (Strava, Inc), which does not accurately represent the general population or even the population of cyclists [28]. The use of preexisting users of digital technologies as the recruitment pool or data set allows for the propagation of disparities in access and uptake of digital technologies. For instance, there are well-established racial imbalances in the uptake of telemedicine [29,30], eHealth record usage [31], and wearables [28]. Indeed, there are deep imbalances by age, rural and urban residency, and socioeconomic status in access to key resources for digital research such as broadband internet [32,33]. This imbalance is even more pronounced when doing research globally [34].

Third, well-conducted digital research can improve the suitability and usability of these technologies for underserved groups. Digital technologies have the capacity to ameliorate or compensate for existing racial, geographic, and socioeconomic disparities. For instance, appropriately deployed and supported telemedicine can improve the management of chronic disease and access to specialist consultations for people in rural or low-mobility populations [9,35]. Efforts to ameliorate racial bias in the accuracy of wearables such as pulse-oximeter accuracy, for example, require recruiting a diverse population during test and validation research [26].

## Existing Frameworks

In the foundational text of research ethics in North America [14], the principle of justice primarily focused on fair subject selection. This focus emphasizes both that individuals are ncluded regardless of race and gender and that an effort is made to balance the burden and benefits of research at the population level [14]:

… even if individual researchers are treating their research subjects fairly, and even if IRBs are taking care to assure that subjects are selected fairly within a particular institution, unjust social patterns may nevertheless appear in the overall distribution of the burdens and benefits of research.

However, the principles of respect for persons and beneficence also acknowledge, at least implicitly, that fairly sharing the burden and benefits of research among the members of a diverse society presents challenges. Respect for persons requires that participants in research are treated as individuals, not simply as members of demographic groups, and that people with diminished autonomy due to cognitive, physical, or social impediments are afforded extra protection. Beneficence requires that the balance of risks and benefits of participation for each individual subject—including vulnerable subjects—is favorable. The Belmont principles thus deal with the difficulties of diversity in research populations by requiring individualized assessment of consent, risks, and benefits for each subject.

In the US context, the Belmont framework is expressed in regulation through the Common Rule [36]. This regulatory framework enshrines additional protections for 3 existing special populations: children, pregnant persons, and prisoners. In addition, it requires researchers to consider the status of any participants who may be “vulnerable to coercion and undue influence,” including military personnel, people who are cognitively disabled, elderly persons, ethnic minorities, refugees, and people who are socioeconomically disadvantaged. While the Common Rule does not specifically advocate for the inclusion of First Nation peoples within research oversight, it does recommend researchers comply with tribal sovereignty, including community-operated institutional review boards [37].

In Canada, the regulatory framework for research is encapsulated in the Tri-Council Policy Statement [38]. Of note, the policy statement explicitly requires an appropriate inclusion of diverse research subjects [38]:

Taking into account the scope and objectives of their research, researchers should be inclusive in selecting participants. Researchers shall not exclude individuals from the opportunity to participate in research on the basis of attributes such as culture, language, religion, race, disability, sexual orientation, ethnicity, linguistic proficiency, gender or age, unless there is a valid reason for the exclusion.

Moreover, there has been a much greater effort to incentivize the careful inclusion of First Nations peoples in research [38]. This is complemented by the OCAP© framework mentioned earlier.

## Challenges of Diversity

### Overview

Broadly representative research populations will be composed of a range of individuals with different capacities, interests, values, and social positions. This poses a problem for attempts to assess the risks and benefits of a particular research design when researchers cannot individualize risk assessment. In particular, researchers cannot assume that the risks and benefits of a study are the same for every individual. We review these differences and the challenges they pose for ethical study design and implementation (Table 1).

**Table 1:** Challenges of research with diverse populations and potential solutions.

Ethical domains and challenges of diverse populations		Potential solutions
**Recruiting representative populations**		
	Different access to technology Different social networks (eg, distinct networks of friends, community groups, and familial connections) Size and cost of study	Follow the principle of "Nothing about us without us" [39] Form community advisory boards and engage with liaisons to help guide recruitment Conduct recruitment alongside community participation and capacity building [40]
**Informed consent**		
	Different understandings of technology Different disclosure practices Translations, including American Sign Language, braille	Multiple information sheets by language Prior engagement toward the cocreation of knowledge Differential practices of risk disclosure that balance informativeness and understanding Targeted resources that enhance the consent process for vulnerable subpopulations
**Secondary use of data**		
	Heightened concerns for data reuse given historical exploitation and data misuse Different expectations about the reuse of data, for example, by age	Explicit notification and assent or withdrawal before deidentification for highly sensitive research Community representation on decision-making bodies
**Privacy**		
	Increased data sensitivity (eg, disability, race, and gender identity) High reidentification risk for hyperminoritized participants Stigmatizing information that may be exacerbated for some subpopulations	Separate demographic markers from primary subject data to prevent reidentification Differential privacy techniques for stigmatizing research Enhanced privacy and confidentiality practices
**Risk to subjects**		
	Specialized data risks to some subjects (eg, undocumented or pregnant persons) Different cognitive capacities to appreciate or absorb risks	Targeted research resources that differentially minimize the risk of participation Inclusion of high-risk participants only as a last resort [41]
**Risk to groups**		
	Stigma for subpopulations generated by incidental research findings (eg, differential rates of alcohol or drug use)	Separate demographic markers from primary subject data
**Benefits to subjects**		
	Different interests (ie, the value of devices and digital services) Different social and technical capacities (ie, internet access and technical platform)	Targeted research resources to equalize the benefits of participation Explicit transfer of data collection technologies to individuals (ie, transfer and training in the use of tablets and laptops at the conclusion of the study)
**Social value of research**		
	Different interests and values Different vulnerabilities to risks of research results (eg, group stigma and state oppression) Limitation of benefits from research (eg, political or economic power)	Explicit analysis of benefits by relevant subgroups Explicit exploration, discussion, and co-created knowledge and transfer to policy for broad and just benefits

### Differential Consent

Individuals may have very different expectations about digital tools and data and require different thresholds and procedures for participant consent. This may be a function of (1) understanding of the scope of participation, (2) tolerance for specific research purposes, and (3) the opportunity to decline participation if data collection occurs as part of routine clinical care or application usage. Each of these may influence the information that a participant requires, how researchers gather consent, and the kinds of research that can be undertaken with a waiver of consent.

Literacy about data collection and reuse is variable. Many individuals may not be aware that data about their physical location or browsing history is routinely collected [42]. Persons with limited experience with digital technologies may have difficulty understanding the capacities, limits, and mechanisms underlying new technologies [43]. Moreover, expectations about the reuse of data may or may not shift depending on whether they are interacting with health care providers, nonprofit organizations, or private commercial entities (eg, Middleton et al [44] on attitudes toward genomic data sharing).

Individuals will have different standards for when risks should be disclosed or consent sought for specific research purposes. For example, in the case of contact tracing using digital apps that involve trade-offs between balancing public benefit and invasion of privacy [45], some individuals may be unwilling to have their deidentified data used if the research would inadvertently stigmatize their racial, gender, or religious group. Thus, while many people may accept deidentified data use for almost all research purposes, some may be particularly sensitive to undisclosed secondary research without their consent. Often marginalized or vulnerable groups may be especially concerned or cautious. Disagreement about research purposes will generate disagreement about what requires specific consent.

If consent to research is incorporated as part of nonresearch interactions (eg, clinical interactions or as part of the license agreement to use a digital service), then different individuals will have different capacities to decline. Access to health services is highly constrained for some groups, and some people may be uncomfortable declining research participation if consent is sought at the point of care. Likewise, awareness of the terms of user agreements to access digital devices and services is notoriously poor [46]. This unequal ability to refuse services complicates the assessment of which consent practices are justifiable.

### Differential Risks and Benefits of Participation

Digital data have a high risk of stigmatizing subjects, placing their employment, income, and immigration status at risk, but these risks are not equal across all groups. In particular, individuals have different vulnerabilities to reidentification and to the harms of confidentiality breaches.

The risk for reidentification [47,48] is particularly high for individuals who are members of a hyperminority (ie, small ethnic subpopulations) or are members of multiple minority classes (ie, intersectionally marginalized). Certain kinds of individual behaviors such as widespread internet usage without privacy-preserving safeguards can also increase the risk of reidentification through commercially available databases, including for noncitizens, women, and youth [49].

Different individuals will have different vulnerability to harm resulting from reidentification. For example, people who are able to become pregnant are subject to special legal risks if reidentification exposes information about their reproductive health [50]. Likewise, individuals with marginalized sexualities or gender identities face differential risk of legal, social, and employment consequences from reidentification depending on their location, social context, and the prevailing legal environment [51-53]. These risks may be hard to assess or control in a deidentified data set that unlinks individuals from their data.

### Differential Social Value

The social value of a study is dependent upon a person’s capacity to access the benefits of the research. Different populations have different abilities to access the products of research, and a study may, therefore, produce insights into a particular condition that does not yield anticipated benefits. The result is a changed risk-benefit calculation for individual community members.

Maximizing social value also requires careful consideration of the different ways individuals assess the risks and benefits of the knowledge generated by research. For example, consider research on early detection of Alzheimer disease using data from mobile devices [54]. False negatives in diagnosis or screening can delay treatment, and false positives can generate unwarranted costs, anxiety, or stigma. Making a fine-grained trade-off between the sensitivity and specificity of a predictor is thus a decision about which people can reasonably disagree. However, it is often difficult to set individualized thresholds for false positives and false negatives. Maximizing social value for a predictor thus requires selecting an appropriate trade-off between adverse events, which differ by severity, frequency, and life impact across different subpopulations.

There are also different levels of vulnerability to the misuse of the research. For example, studies using digital tools (ie, social media data analysis) may generate incidental findings about the association between negatively valenced behaviors (such as suicidality, drug consumption, and negative moods) and marginalized racial groups, religions, sexualities, or genders. Moreover, the risk is asymmetric: the findings that run contrary to invidious stereotypes may not undermine popular beliefs in those stereotypes, even as findings, which support the stereotype reinforce the popular belief [55]. Marginalized communities suffer more from research that is potentially stigmatizing because they may start with fewer resources and opportunities.

## Traditional Principles in the Context of Diversity

Traditional principles are workable in a context where the research population is relatively homogenous—where risks and benefits are similar or easy to calculate, where the information required to consent is easily accessible, and where values and mental models are uniform. With variability on any of the three dimensions, problems may appear:

1. Appropriately informing participants becomes more difficult as the vulnerabilities, languages, cultural understandings, and mental models of technology become more diverse. Diversity generates a dilemma for the information provision function of informed consent, by requiring investigators to either: (i) specify the full range of risks to which any member of the research population could reasonably be exposed or (ii) tailor information materials and procedures for each sufficiently distinct subgroup [56,57]. In the first strategy, the autonomy of subjects may be undermined by risk information that is irrelevant to their circumstances, potentially overloading the capacity of some individuals to meaningfully digest or understand risks. In the second strategy, the burden upon researchers becomes more significant as the diversity of the group increases. More importantly, providing different information to subjects enrolled in the same study may violate respect for persons. Moreover, if researchers elide or simplify risks to a subject group because they believe the subjects are, on average, unlikely to be exposed to the risk, there is a serious risk that they will fail to fully inform members of that group [58].
2. Calculating the risk-benefit ratio for subjects becomes more difficult as the vulnerabilities, interests, and values of research participants become more diverse. According to the prevailing view in research ethics, some subjects should be excluded from studies, even if they would otherwise consent, if their participation would pose a sufficiently grave risk to their well-being. As the overlapping dimensions of diversity increase, so does the risk that at least some subjects are at high enough risk that they ought to be excluded [59]. Even innocuous data collection can place some members of a diverse research population at risk. For instance, the participation of undocumented persons [60], gender-diverse [52], or pregnant persons in seemingly innocuous research may expose location, financial, or health care data in ways that place them at risk of legal or social sanction in some jurisdictions. As diversity increases, however, it may become increasingly difficult to identify these individuals through simple demographic cues nor may participants feel comfortable reporting their status to researchers.
3. Calculating the social value of the research for the broader population requires considering the wide variety of different interests, religious and moral commitments, and material circumstances present within a plural society. Research impacts not only direct participants but also those who pay for, consume, benefit from, and live with the consequences of the research [15]. The social value requirement is sometimes understood as a minimal requirement that research has the potential to contribute valuable knowledge [61], while others view it as a more stringent requirement that research contributes to the maintenance of a just society [20]. At the very least, the social value of a research study cannot be thought of as a monolith: in a plural society, the costs and benefits of research will be different for different subpopulations [15]. If this is true, then the ethics of a research project must attend to the ways that research benefits and harms are distributed unequally. This is true not only for digital research with diverse populations but for all digital research.

## Reorienting Toward Justice

The transactional model of researcher-participant ethics has created many of the challenges described so far: diverse research populations are addressed through individualized assessments that require individual consent and favorable individual risk-benefit. If any of these features fail, researchers are inclined to exclude individuals from a study. The rationales offered are:

1. Individualized consent is labor intensive, and subject diversity increases the risk that standardized consent practices will not adequately inform all participants. Researchers who wish to keep consent procedures manageable thus face a dilemma between, on the one hand, underdescribing research risks for some subjects and, on the other hand, overwhelming subjects with difficult-to-understand information.
2. While diversity raises the risk that at least some individuals will be harmed by participation, excluding those individuals because of their different risk profile may diminish the representativeness of data. Hence, there appears to be a trade-off between maximally protecting each individual subject and creating inclusive research projects that yield knowledge of benefit to a diverse society [62].
3. Some research designs, such as secondary reuse, do not permit individualized risk assessment or consent practices. Many risks arise in the context of deidentified secondary reuse, where (i) individuals can be reidentified, especially if in a minority population and (ii) where population-level findings can harm minoritized populations. Yet, these kinds of research do not allow for the individualized exclusion of subjects, and hence risks must be managed at the population level.

A complementary or alternative model is required. A reorientation of digital research ethics to emphasize social justice is supported by an overlapping consensus between recent work that explicitly considers the broader social and institutional ramifications of research. London [19] and Wenner [20] have argued that research is a system of social cooperation rather than a transaction between participant and researcher. Research should instead be understood as a collective activity that individuals engage in to protect and promote their basic interests through the shared production of a unique public good (ie, research knowledge). Likewise, the concept of data solidarity has been proposed by Prainsack et al [63] to emphasize collective control over research—private or public—to ensure that data are used in the public interest. This contrasts with the transactional model, which relies on a form of data altruism from subjects that treat digital data collection as a gift by subjects to researchers and fail to acknowledge the power imbalances between data subjects and data collectors, the harms that research can do to nonsubjects, and the potential for private profiteering from public data sets. Finally, participatory research methods reimagine participants as coresearchers, enabling the creation of new knowledge while meaningfully building capacity in affected communities [64]. These methods have been operationalized by indigenous and First Nations communities through the CARE framework and the OCAP principles, which focus on community control and capacity building beyond the direct subjects of the research.

These existing approaches adopt the tools of political philosophy to argue that research should not merely respect individual participants but also ensure a just allocation of the benefits and burdens of research within the community as a whole. While we cannot provide a full justification of these approaches in this paper, a way of specifying rights and obligations with respect to research is to consider the principles of research ethics to a community would agree to if they were unaware of their existing social roles, talents, wealth, or substantive moral commitments [65]. This contractarian approach to justice has a venerable history among moral and political philosophers [65-67] and has been articulated in the context of health care justice [68] and research ethics [19,20]. On this view, the rules governing digital research would be structured so that the production of research knowledge protects the basic interests shared by all individuals in society, with special attention paid to the flourishing of the least advantaged members of society. This agreement would specify (1) the procedures to protect the autonomy of each individual to control participation in digital research, (2) general principles for fairly distributing the benefits and burdens of digital research, where deviations from a roughly equal distribution are permissible only if they are to the advantage of the most vulnerable, (3) a method for ensuring the legitimacy of the research and allowing for participants and affected community members to exercise control over crucial features of the research, and (4) that if the proceeding conditions are met, then there is a collective obligation to engage in the production of knowledge that serves to create a society that enables the flourishing of all people regardless of their advantages or disadvantages. While this does not yet specify concrete practices, it provides an alternative moral framework to the Belmont principles for justifying the development of guidelines for digital research with diverse populations.

## Specific Recommendations

### Overview

Drawing on this existing work, we outline considerations for good practices upon which researchers can draw and that will promote trust in the digital research process across diverse participant populations. While individual researchers cannot make the institutional and regulatory changes required to align with a justice-first view of digital research ethics, we believe that individual researchers can and should begin to attend to the special challenges of working with diverse study populations. The following considerations suggest that the digital research community should collectively work toward a fuller specification of justice that translates the existing work into the specific modalities of digital research with diverse populations.

### Protect Individual Autonomy in High-Risk Situations

Digital research often exposes different subjects to different autonomy, privacy, and reputational risks. These are not well mitigated by standard collection and consent procedures, and the risks themselves are often invisible to researchers. As Prainsack et al [63] point out, as the risks to individuals increase or the public value of the research diminishes, collective control of data sets and individual rights to refuse should be strengthened. Researchers should thus consider the following practices:

1. Use a community advisory board and community liaisons throughout the study design process to identify high-risk activities and subpopulations requiring extra review and tailored consent processes.
2. Implement linguistically tailored consent processes for sufficient large or sufficiently vulnerable subpopulations. While this may increase the burden on researchers and oversight boards to vet additional consent documents, it is an important method for showing respect for the participation of individuals who do not primarily communicate in the dominant language of the research population.
3. When engaging with people who hold divergent prior understandings of technology, prepare multiple risk disclosure strategies to balance informativeness and understanding and target study resources to enhance the consent process for more vulnerable subpopulations.
4. Explore institutional mechanisms and regulatory changes to allow for recontact to allow opportunities for withdrawal, including from secondary research with high risks for reidentification or community stigmatization. Given the privacy risks associated with maintaining data links to participants to enable recontact, the continued development of technical and sociotechnical practices for the preservation of participant privacy is essential [69].

### Fairly Distribute Benefits and Burdens

Including a more diverse research population is an important method for a wide distribution of the benefits of research but may also burden already disadvantaged groups. While researchers cannot eliminate structural injustices completely, they should compensate for them in the design and conduct of their studies where possible. While there is likely to be disagreement over what methods of compensation are fair, a widely endorsed approach is to embrace methods that are to the benefit of the least advantaged members of society. This may include the following:

1. Target research resources to differentially minimize uneven risks of participation and barriers to research access. This may include capacity building among participating communities (eg, knowledge building around digital technologies).
2. In research with a high risk of reidentification or stigmatization, take care to enhance subject and community privacy. This may include differential privacy techniques [69] and the separation of demographic markers from primary subject data to prevent the reidentification of individuals and incidental stigmatization of marginalized populations.
3. Target research resources that equalize the benefits of participation for different groups with sensitivity to their different needs and capacities. Be explicit and proactive in developing mechanisms for disseminating results and policy mechanisms to ensure broad benefits of resulting knowledge.

### Embrace Transparency and Build Collective Control of Research

Research often requires value judgments, and there may be widespread disagreement between researchers, participants, and the broader community over which values to promote. While resolving this disagreement is not simple, at the very least, it requires researchers to be transparent about their purposes, methods, and results. More controversially, but in alignment with the ethos of participatory research methods, it may require institutional mechanisms for community members to control the direction of the research, invigilate risks, and ultimately steward data sets. This requires that researchers:

1. Clearly report research ethics methodologies, including specifying methods of consent, review of risks to participants, reporting of adverse events, and expected costs and benefits of producing the knowledge [3,70,71].
2. Incorporate community liaisons as members of the study team to identify connections with prospective participants and populations, tailoring consent documents, meaningful data analysis, and capacity building.
3. Educate and empower community advisory boards, especially for high-risk studies, to share in study design or development of study protocols.
4. Consider mechanisms for community ownership of data sets and strategies for limiting access to secondary reuse if deemed appropriate by community advisory boards or participants’ representatives [12].

Implementation of all of these practices requires time, expertise, and money. The number of strategies that will need to be adopted is proportional to the expected balance of benefits and harms to participants and their communities [72]. The goal of doing research well, in ways that honor the trust that communities place in researchers, is non-negotiable.

## Conclusions

The explicit integration of justice in digital research recognizes that societies have a collective duty to help produce valuable knowledge under fair terms of cooperation. While more work needs to be done to fully explicate the institutional changes and individual practices that will realize justice in digital research, existing work in participatory action research, neuroethics, political philosophy, and indigenous research methods already provide an invaluable foundation for discharging this obligation.

## Abbreviations

CARE: Collective benefit, Authority to Control, Responsibility, and Ethics
OCAP: ownership, control, access, and possession
